# Supplementary material for: Discovery and application of insertion-deletion (INDEL) polymorphisms for QTL mapping of early life-history traits in Atlantic salmon
Source: BMC Genomics. 2010 Mar 8;11:156. doi: 10.1186/1471-2164-11-156 (PMC2838853; doi:10.1186/1471-2164-11-156)
Supplement: Additional file 2 — Information on developed 76 locus single-run INDEL panel in Atlantic salmon. Information on fluorescence labeling, primer concentrations, PCR pooling and links to alignments, INDEL motifs and GENESCAN (Burge and Karlin 1997) predictions of genes/exons are available in html format. [file 1471-2164-11-156-S2.ZIP › Additionalfile2/table13587.html]

Contig Alignment

```
Base	A B C D E F   
1	G . . . G .    
2	A . . . A .    
3	G . . . G .    
4	T . . . T .    
5	G . . . G .    
6	C . . . C .    
7	A . . . A .    
8	G . . . G .    
9	T . . . T .    
10	G . . . G .    
11	A . . . A .    
12	G . . . G .    
13	A . . . A .    
14	T . . . T .    
15	A . . . A .    
16	T . . . T .    
17	T . . . T .    
18	T . . . T .    
19	T . . . T .    
20	T . . . T .    
21	A . . . A .    
22	T . . . T .    
23	T . . . T .    
24	T . . . T .    
25	C . . . C .    
26	G . . . G .    
27	G . . . G .    
28	G . . . G .    
29	A . . . A .    
30	A . . . A .    
31	A . . . A .    
32	T . . . T .    
33	A A A A A .    
34	G G G G G .    
35	T T T T T .    
36	G G G G G .    
37	C C C C C .    
38	T T T T T .    
39	T T T T T .    
40	T T T T T .    
41	T T T T T .    
42	G G G G G .    
43	T T T T T .    
44	C C C C C .    
45	- A A A - .  2	 indel  
46	- C C C - .  2	 indel  
47	A A A A A .    
48	G G G G G .    
49	A A A A A .    
50	G G G G G .    
51	A A A A A .    
52	C C C C C .    
53	T T T T T .    
54	T T T T T .    
55	A A A A A .    
56	T T T T T .    
57	T T T T T .    
58	T T T T T .    
59	G G G G G .    
60	A A A A A .    
61	C C C C C .    
62	T T T T T T    
63	T T T T T T    
64	A A A A A A    
65	T T T T T T    
66	C C C C C C    
67	T T T T T T    
68	T T T T T T    
69	C C C C C C    
70	C C C C C C    
71	A A A A A A    
72	A A A A A A    
73	A A A A A A    
74	T T T T T T    
75	G G G G G G    
76	T T T T T T    
77	T T T T T T    
78	A A A A A A    
79	A A A A A A    
80	A A A A A A    
81	A A A A A A    
82	C C C C C C    
83	A A A A A A    
84	G G G G G G    
85	G G G G G G    
86	A A A A A A    
87	G G G G G G    
88	T T T T T T    
89	A A A A A A    
90	G G G G G G    
91	C C C C C C    
92	C C C C C C    
93	A A A A A A    
94	A A A A A A    
95	T T T T T T    
96	C C C C C C    
97	T T T T T T    
98	T T T T T T    
99	C C C C C C    
100	C C C C C C    
101	T T T T T T    
102	C C C C C C    
103	C C C C C C    
104	T T T T T T    
105	G G G G G G    
106	G G G G G G    
107	A A A A A A    
108	G G G G G G    
109	A A A A A A    
110	G G G G G G    
111	T T T T T T    
112	T T T T T T    
113	A A A A A A    
114	C C C C T T  2	 transition  
115	T T T T T T    
116	G G G G G G    
117	G G G G G G    
118	G G G G G G    
119	T T T T T T    
120	A A A A A A    
121	A A A A A A    
122	C C C C C C    
123	C C C C C C    
124	T T T T T T    
125	C C C C C C    
126	G G G G G G    
127	T T T T T T    
128	A A A A A A    
129	C C C C C C    
130	C C C C C C    
131	C C C C C C    
132	A A A A A A    
133	G G G G G G    
134	T T T T T T    
135	A A A A A A    
136	C C C C C C    
137	C C C C C C    
138	C C C C C C    
139	A A A A A A    
140	A A A A A A    
141	T T T T T T    
142	A A A A A A    
143	C C C C C C    
144	G G G G G G    
145	T T T T T T    
146	T T T T T T    
147	T T T T T T    
148	T T T T T T    
149	T T T T T T    
150	G G G G G G    
151	T T T T T T    
152	T T T T T T    
153	C C C C C C    
154	C C C C C C    
155	A A A A A A    
156	G G G G G G    
157	C C C C C C    
158	T T T T T T    
159	C C C C C C    
160	T T T T T T    
161	G G G G G G    
162	C C C C C C    
163	T T T T T T    
164	C C C C C C    
165	T C T T T T    
166	A A A A A A    
167	A A A A A A    
168	C C C C C C    
169	A A A A A A    
170	C C C C C C    
171	A A A A A A    
172	T T T T T T    
173	C C C C C C    
174	T T T T T T    
175	T T T T T T    
176	G G G G G G    
177	A A A A A A    
178	C C C C C C    
179	A A A A A A    
180	T T T T T T    
181	T T T T T T    
182	G G G G G G    
183	A A A A A A    
184	T T T T T T    
185	T T T T T T    
186	A A A A A A    
187	G G G G G G    
188	C C C C C C    
189	C C C C C C    
190	G G G G G G    
191	A A A A A A    
192	A A A A A A    
193	T T T T T T    
194	C C C C C C    
195	A A A A A A    
196	G G G G G G    
197	G G G G G G    
198	T T T T T T    
199	G G G G G G    
200	T T T T T T    
201	G G G G G G    
202	T T T T T T    
203	T T T T T T    
204	A A A A A A    
205	G G G G G G    
206	A A A A A A    
207	G G G G G G    
208	C C C C C C    
209	A A A A A A    
210	G G G G G G    
211	G G G G G G    
212	G G G G G G    
213	C C C C C C    
214	T T T T T T    
215	A A A A A A    
216	G G G G G G    
217	A A A A A A    
218	A A A A A A    
219	C C C C C C    
220	A A A A A A    
221	A A A A A A    
222	G G G G G G    
223	A A A A A A    
224	C C C C C C    
225	T T T T T T    
226	A A A A A A    
227	C C C C C C    
228	A A A A A A    
229	T T T T T T    
230	A A A A A A    
231	G G G G G G    
232	G G G G G G    
233	C C C C C C    
234	C T T T T T    
235	A A A A A A    
236	C C C C C C    
237	C C C C C C    
238	C C C C C C    
239	A A A A A A    
240	G G G G G G    
241	T T T C T T    
242	T T T T T T    
243	G G G G G G    
244	C C C C C C    
245	T T T T T T    
246	C C C C C C    
247	T T T T T T    
248	A A A A A A    
249	C T T T T T    
250	A A A A A A    
251	G G G G G G    
252	G G G G G G    
253	A A A A A A    
254	G G G G G G    
255	G G G G G G    
256	A A A A A A    
257	T T T T T T    
258	A A A A A A    
259	T T T T T T    
260	T T T T T T    
261	A A A A A A    
262	T T T T T T    
263	A A A A A A    
264	A A A A A A    
265	T T T T T T    
266	T T T T T T    
267	A A A A A A    
268	G G G G G G    
269	C C C C C C    
270	A A A A A A    
271	G G G G G G    
272	A A A A A A    
273	T T T T T T    
274	T T T T T T    
275	A A A A A A    
276	T T T T T T    
277	A A A A A A    
278	A A A A A A    
279	A A A A A A    
280	T T T T T T    
281	A A A A A A    
282	A A A A A A    
283	T T T T T T    
284	T T T T T T    
285	T T T T T T    
286	G G G G G G    
287	T T T T T T    
288	A A A A A A    
289	G G G G G G    
290	A A A A A A    
291	T T T T T T    
292	A A A A A A    
293	A A A A A A    
294	A A A A A A    
295	A A A A A A    
296	T T T T T T    
297	A A A A A A    
298	A A A A A A    
299	T T T T T T    
300	G G G G G G    
301	T A T T T T    
302	T T T T T T    
303	A A A A A A    
304	A A A A A A    
305	A A A A A A    
306	A A A A A A    
307	G G G G G G    
308	G G G G G G    
309	T T T T T T    
310	T T T T T T    
311	C C C C C C    
312	A A A A A A    
313	T T T T T T    
314	T T T T T T    
315	A A A A A A    
316	T T T T T T    
317	A A A A A A    
318	T T T T T T    
319	G G G G G G    
320	T T T T T T    
321	A A A A A A    
322	T T T T T T    
323	A A A A A A    
324	A A A A A A    
325	A A A A A A    
326	T T T T T T    
327	T T T T T T    
328	T T T T T T    
329	A A A A A A    
330	A A A A A A    
331	A A A A A A    
332	A A A A A A    
333	C C C C C C    
334	A A A A A A    
335	T T T T T T    
336	A A A A A A    
337	C C C C C C    
338	T T T T T T    
339	G G G G G G    
340	G G G G G G    
341	T T T T T T    
342	G G G G G G    
343	A A A A A A    
344	T T T T T T    
345	G G G G G G    
346	A A A A A A    
347	T T T T T T    
348	C C C C C C    
349	T T T T T T    
350	T T T T T T    
351	A A A A A A    
352	A A A A A A    
353	A A A A A A    
354	A A A A A A    
355	G G G G G G    
356	G G G G G G    
357	A A A A A A    
358	G G G G G G    
359	T T T T T T    
360	A A A A A A    
361	T T T T T T    
362	A A A A A A    
363	T T T T T T    
364	A A A A A A    
365	C C C C C C    
366	C C C C C C    
367	A A A A A A    
368	A A A A A A    
369	A A A A A A    
370	C C C C C C    
371	A A A A A A    
372	C C C C C C    
373	T T T T T T    
374	T T T T T T    
375	T T T T T T    
376	C C C C C C    
377	A A A A A A    
378	T T T T T T    
379	A A A A A A    
380	C C C C C C    
381	A A A A A A    
382	A A A A A A    
383	A A A A A A    
384	A A A A A A    
385	T T T T T T    
386	A A A A A A    
387	A A A A A A    
388	C C C C C C    
389	A A A A A A    
390	G G G G G G    
391	A A A A A A    
392	A A A A A A    
393	A A A A A A    
394	A A A A A A    
395	T T T T T T    
396	A A A A A A    
397	T T T T T T    
398	G G G G G G    
399	C C C C C C    
400	T T T T T T    
401	C C C C C C    
402	C C C C C C    
403	T T T T T T    
404	C C C C C C    
405	C C C C C C    
406	C C C C C C    
407	T T T T T T    
408	T T T T T T    
409	A A A A A A    
410	T T T T T T    
411	T T T T T T    
412	C C C C C C    
413	C C C C C C    
414	T T T T T T    
415	C C C C C C    
416	C C C C C C    
417	A A A A A A    
418	T T T T T T    
419	T T T T T T    
420	G G G G G G    
421	T T T T T T    
422	G G G G G G    
423	A A A A A A    
424	T T T T T T    
425	T T T T T T    
426	A A A A A A    
427	C C C C C C    
428	A A A A A A    
429	G G G G G G    
430	T T T T T T    
431	A A A A A A    
432	C C C C C C    
433	A A A A A A    
434	A A A A A A    
435	T T T T T T    
436	T T T T T T    
437	A A A A A A    
438	A A A A A A    
439	T T T T T T    
440	C C C C C C    
441	A A A A A A    
442	A A A A A A    
443	T T T T T T    
444	C C C C C C    
445	A A A A A A    
446	T T T T T T    
447	C C C C C C    
448	A A A A A A    
449	C C C C C C    
450	A A A A A A    
451	A A A A A A    
452	C C C C C C    
453	T T T T T T    
454	T T T T T T    
455	C C C C C C    
456	T T T T T T    
457	C C C C C C    
458	A A A A A A    
459	G G G G G G    
460	A A A A A A    
461	G G G G G G    
462	G G G G G G    
463	C C C C C C    
464	C C C C C C    
465	T T T T T T    
466	G G G G G G    
467	G G G G G G    
468	G G G G G G    
469	A A A A A A    
470	A A A A A A    
471	G G G G G G    
472	A A A A A A    
473	G G G G G G    
474	G G G G G G    
475	T T T T T T    
476	A A A A A A    
477	G G G G G G    
478	A A A A A A    
479	A A A A A A    
480	G G G G G G    
481	T T T T T T    
482	G G G G G G    
483	C C C C C C    
484	T T T T T T    
485	T T T T T T    
486	T T T T T T    
487	G G G G G G    
488	T T T T T T    
489	T T T T T T    
490	A A A A A A    
491	G G G G G G    
492	A A A A A A    
493	G G G G G G    
494	T T T T T T    
495	G G G G G G    
496	G G G G G G    
497	C C C C C C    
498	T T T T T T    
499	C C C C C C    
500	T T T T T T    
501	G G G G G G    
502	T T T T T T    
503	C C C C C C    
504	T T T T T T    
505	T T T T T T    
506	T T T T T T    
507	G G G G G G    
508	G G G G G G    
509	T T T T T T    
510	C C C C C C    
511	G G G G G G    
512	G G G G G G    
513	C C C C C C    
514	A A A A A A    
515	T T T T T T    
516	C C C C C C    
517	T T T T T T    
518	G G G G G G    
519	T T T T T T    
520	C C C C C C    
521	T T T T T T    
522	G G G G G G    
523	G G G G G G    
524	G G G G G G    
525	G G G G G G    
526	A A A A A A    
527	A A A A A A    
528	A A A A A A    
529	T T T T T T    
530	C C C C C C    
531	T T T T T T    
532	T T T T T T    
533	C C C C C C    
534	A A A A A A    
535	A A A A A A    
536	A A A A A A    
537	G G G G G G    
538	C C C C C C    
539	- C - - - -    
540	G G G G G G    
541	T T T T T T    
542	C C C C C C    
543	C C C C C C    
544	C C C C C C    
545	A A A A A A    
546	T T T T T T    
547	G G G G G G    
548	A A A A A A    
549	C C C C C C    
550	C C C C C C    
551	T T T T T T    
552	T T T T T T    
553	T T T T T T    
554	C C C C C C    
555	T T T T T T    
556	G G G G G G    
557	A A A A A A    
558	A A A A A A    
559	C C C C C C    
560	A A A A A A    
561	A A A A A A    
562	T T T T T T    
563	T T T T T T    
564	C C C C C C    
565	T T T T T T    
566	T T T T T T    
567	C C C C C C    
568	T T T T T T    
569	A A A A A A    
570	T T T T T T    
571	A A A A A A    
572	T T T T T T    
573	G G G G G G    
574	T A A A T T  3	 transversion  
575	T T T T T T    
576	C C C C C C    
577	A A A A A A    
578	T T T T T T    
579	C C C C C C    
580	A A A A A A    
581	C C C C C C    
582	A A A A A A    
583	G G G G G G    
584	G G G G G G    
585	C C C C C C    
586	A A A A A A    
587	T T T T T T    
588	T T T T T T    
589	G G G G G G    
590	G . G G G G    
591	G . T T T T    
592	. . G G G G    
593	. . T T T T    
594	. . T T T T    
595	. . G G G G    
596	. . A A A A    
597	. . A A A A    
598	. . T T T T    
599	. . A A A A    
600	. . T T T T    
601	. . C C C C    
602	. . T T T T    
603	. . C C C C    
604	. . C C C C    
605	. . A A A A    
606	. . T T T T    
607	. . G G G G    
608	. . A A A A    
609	. . T T T T    
610	. . G G G G    
611	. . C C C C    
612	. . G G G G    
613	. . T T T T    
614	. . T T T T    
615	. . G G G G    
616	. . G G G G    
617	. . A A A A    
618	. . G G G G    
619	. . A A A A    
620	. . A A A A    
621	. . A A A A    
622	. . A A A A    
623	. . A A A A    
624	. . G G G G    
625	. . G G G G    
626	. . G G G G    
627	. . T T T T    
628	. . G G G G    
629	. . C C C C    
630	. . C C C C    
631	. . C C C C    
632	. . A A A A    
633	. . A A A A    
634	. . C C C C    
635	. . T T T T    
636	. . T T T T    
637	. . T T T T    
638	. . G G G G    
639	. . G G G G    
640	. . A A A A    
641	. . T T T T    
642	. . C C C C    
643	. . T T T T    
644	. . C C C C    
645	. . T T T T    
646	. . G G G G    
647	. . . T T T    
648	. . . A A A    
649	. . . G G G    
650	. . . G G G    
651	. . . A A A    
652	. . . C C C    
653	. . . A A A    
654	. . . C C C    
655	. . . A A A    
656	. . . G G G    
657	. . . T T T    
658	. . . A A A    
659	. . . T T T    
660	. . . C C C    
661	. . . T T T    
662	. . . G G G    
663	. . . G G G    
664	. . . T T T    
665	. . . G G G    
666	. . . T T T    
667	. . . G G G    
668	. . . C C C    
669	. . . A A A    
670	. . . G G G    
671	. . . G G G    
672	. . . A A A    
673	. . . G G G    
674	. . . A A A    
675	. . . G G G    
676	. . . G G G    
677	. . . A A A    
678	. . . G G G    
679	. . . G G G    
680	. . . G G G    
681	. . . A A A    
682	. . . G G G    
683	. . . A A A    
684	. . . T T T    
685	. . . G G G    
686	. . . A A A    
687	. . . A A A    
688	. . . G G G    
689	. . . T T T    
690	. . . C C C    
691	. . . C C C    
692	. . . T T T    
693	. . . T T T    
694	. . . C C C    
695	. . . T T T    
696	. . . C C C    
697	. . . T T T    
698	. . . T T T    
699	. . . T T T    
700	. . . G G G    
701	. . . T T T    
702	. . . G G G    
703	. . . C C C    
704	. . . T T T    
705	. . . . C C    
706	. . . . C C    
707	. . . . C C    
708	. . . . T T    
709	. . . . C C    
710	. . . . C C    
711	. . . . T T    
712	. . . . G G    
713	. . . . A A    
714	. . . . T T    
715	. . . . T T    
716	. . . . G G    
717	. . . . A A    
718	. . . . G G    
719	. . . . T T    
720	. . . . C C    
721	. . . . A A    
722	. . . . C C    
723	. . . . T T    
724	. . . . C C    
725	. . . . T T    
726	. . . . C C    
727	. . . . A A    
728	. . . . A A    
729	. . . . A A    
730	. . . . C C    
731	. . . . C C    
732	. . . . C C    
733	. . . . C C    
734	. . . . A A    
735	. . . . G G    
736	. . . . G G    
737	. . . . T T    
738	. . . . C C    
739	. . . . A A    
740	. . . . A A    
741	. . . . G G    
742	. . . . G G    
743	. . . . C C    
744	. . . . T T    
745	. . . . G G    
746	. . . . G G    
747	. . . . A A    
748	. . . . C C    
749	. . . . C C    
750	. . . . C C    
751	. . . . A A    
752	. . . . G G    
753	. . . . G G    
754	. . . . C C    
755	. . . . A A    
756	. . . . C C    
757	. . . . T T    
758	. . . . T T    
759	. . . . T T    
760	. . . . G G    
761	. . . . T T    
762	. . . . C C    
763	. . . . A A    
764	. . . . C C    
765	. . . . T T    
766	. . . . G G    
767	. . . . A A    
768	. . . . C C    
769	. . . . C C    
770	. . . . C C    
771	. . . . T T    
772	. . . . C C    
773	. . . . G G    
774	. . . . T T    
775	. . . . G G    
776	. . . . C C
```
